# Lipid-Induced Mechanisms of Metabolic Syndrome

**DOI:** 10.1155/2020/5762395

**Published:** 2020-08-26

**Authors:** Yulia K. Denisenko, Oxana Yu Kytikova, Tatyana P. Novgorodtseva, Marina V. Antonyuk, Tatyana A. Gvozdenko, Tatyana A. Kantur

**Affiliations:** ^1^Vladivostok Branch of the Far Eastern Scientific Centre of Physiology and Pathology of Respiration, Institute of Medical Climatology and Rehabilitative Treatment, Vladivostok 690105, Russia; ^2^Far Eastern Federal University, Vladivostok 690950, Russia

## Abstract

Metabolic syndrome (MetS) has a worldwide tendency to increase and depends on many components, which explains the complexity of diagnosis, approaches to the prevention, and treatment of this pathology. Insulin resistance (IR) is the crucial cause of the MetS pathogenesis, which develops against the background of abdominal obesity. In light of recent evidence, it has been shown that lipids, especially fatty acids (FAs), are important signaling molecules that regulate the signaling pathways of insulin and inflammatory mediators. On the one hand, the lack of n-3 polyunsaturated fatty acids (PUFAs) in the body leads to impaired molecular mechanisms of glucose transport, the formation of unresolved inflammation. On the other hand, excessive formation of free fatty acids (FFAs) underlies the development of oxidative stress and mitochondrial dysfunction in MetS. Understanding the molecular mechanisms of the participation of FAs and their metabolites in the pathogenesis of MetS will contribute to the development of new diagnostic methods and targeted therapy for this disease. The purpose of this review is to highlight recent advances in the study of the effect of fatty acids as modulators of insulin response and inflammatory process in the pathogenesis and treatment for MetS.

## 1. Introduction

Metabolic syndrome (MetS) is a complex of several disorders (abdominal obesity, hyperglycemia, hypertriglyceridemia, and hypertension), which together dramatically raise the risk of developing atherosclerotic cardiovascular disease, insulin resistance, and diabetes mellitus [1, 2]. Because the prevalence of obesity has doubly increased worldwide over the past 30 years, the prevalence of MetS has markedly boosted in tandem [2–5]. Currently, clinicians and researchers have not identified an optimal treatment for MetS, and consequently, it is critical to identify new ways of approaching this syndrome in order to identify efficacious methods of diagnosing, screening, and treating MetS. Most researchers believe that hyperinsulinemia and/or insulin resistance (IR) is the first link in the chain of clinical-metabolic disturbances of MetS [5–7]. The development of IR is the result of a long chain of pathological events. Lipids play the crucial role in the pathogenesis of IR and the subsequent development of MetS [8–18]. All lipids are no longer considered the same. It is well known that excessive consumption of saturated fats contributes to the development of obesity and related diseases [19]. It has now been shown that high plasma levels of free fatty acids (FFAs), particularly saturated fatty acids (SFAs), may be associated with insulin resistance in obese patients with type 2 diabetes mellitus [17]. The lack of polyunsaturated fatty acids (PUFAs), especially n-3 PUFAs, some phospholipids, and plasmalogens in the cell membrane, is the cause of changes in glucose-insulin homeostasis and the development of inflammation [10, 13, 20–23]. Conversely, multiple investigations have established a connection between inflammation and changes in lipids and their derivatives in the setting of MetS [24–29]. Alteration in the metabolism of fatty acids affects the synthesis of eicosanoids and pro-resolving lipid mediators responsible for immune-metabolic homeostasis [30–33]. Recent studies have further elucidated the role of these metabolites in the contribution to the chronic, low-grade inflammatory state in MetS [34–36]. A comprehensive understanding of the importance of lipids in the pathogenesis of MetS contributes to the development of preventive and targeted lipid-correcting therapy. The aim of the review is to analyse the modern views on the role of lipids, particularly PUFAs and FFAs, in the pathogenesis of MetS. In this review, we summarized the molecular mechanisms of the relationship between fatty acids and glucose transport, inflammatory response, mitochondrial dysfunction, and endoplasmic reticulum stress in the development of MetS.

## 2. Metabolic Syndrome: Definitions and Criteria

Metabolic syndrome (MetS) has become a widely debated scientific, medical, and social problem worldwide. Indeed, the definition of metabolic syndrome is important for clinical practice and deserves serious scientific and medical research. MetS is characterized by the following clinical criteria: abdominal obesity, decreased peripheral tissue sensitivity to insulin, and hyperinsulinemia, which cause metabolic disorders of carbohydrates, lipids, and purines [1, 2]. This combination of metabolic disorders is often found in one person and, thus, significantly increases the risk of cardiovascular disease (CVD), type 2 diabetes mellitus (T2DM), arthritis, chronic kidney disease, schizophrenia, nonalcoholic fatty liver disease (NAFLD), and several types of cancer [37–42].

MetS is characterized by a steadily increasing prevalence [3, 4]. However, its prevalence rates vary depending on the criteria used to determine MetS, genetic component, gender, age, population and area of residence, education, level of physical activity, nutrition, and lifestyle [39]. Approximately one-fourth of world's adult population have MetS [43, 44]. Urbanization and its associated sedentary lifestyle and surplus nutrition are the root cause of this global epidemic.

The determination of MetS uses the criteria of the following medical communities: WHO (World Health Organization), NCEPATP III (National Cholesterol Education Program-Adult Treatment Panel III), AACE (American Association of Clinical Endocrinologists), IDF (International Diabetes Federation), EGIR (European Group for the Study of Insulin Resistance), The International Diabetes Federation (IDF), American Heart Association/National Heart, Lung and Blood Institute (AHA/NLHBI), World Heart Federation (WHF), International Atherosclerosis Society (IAS), and The International Association for the Study of Obesity (IASO) [3]. A guideline was made in 2009 to unify the criteria for the diagnosis of MеtS. According to this guide, three of the five criteria are necessary for diagnosing MetS: (1) waist circumference ≥102 cm for males and ≥88 cm for females (for Asians ≥90 cm for males and ≥80 cm for females); (2) systolic blood pressure ≥130 mmHg or diastolic blood pressure ≥85 mmHg or antihypertensive medication; (3) fasting plasma glucose ≥5.6 mmol/L or on medication for high blood glucose; (4) HDL cholesterol <1.03 mmol/L for males and <1.30 mmol/L for females or on medications for reduced HDL cholesterol; and (5) triglycerides ≥1.7 mmol/L or on medications for elevated triglycerides [3].

Although the exact etiology of the MetS is not clearly understood, insulin resistance (IR) is considered as the principal factor for the pathogenesis of this syndrome [6, 11, 18, 45]. As found by the insulin-modified, frequently sampled intravenous glucose tolerance assay, insulin sensitivity is significantly lower in patients with two or more components of the MetS compared to those with none of these components [2]. Dysregulation of lipid metabolism is considered as an important link in the overall development chain of IR. It is well known that lipids play a critical role in the regulation of energy metabolism, glucose transport, and immune process in many organs and tissues such as the liver, adipose tissue, muscle, heart, and gastrointestinal tract [46]. However, the molecular mechanisms of this regulation remain largely unexplored. The study of lipid metabolism disorders is a promising direction for the development of methods for the effective treatment of this pathology.

## 3. Polyunsaturated Fatty Acids and Metabolic Syndrome Risk

Fatty acids (FAs) play multiple roles in humans and other organisms. Most importantly, FAs are a substantial part of lipids. Fatty acids are either saturated or unsaturated carboxylic acids with carbon chains varying between 2 and 36 carbon atoms. Polyunsaturated fatty acids with an acid end containing the functional carboxylic acid group and a methyl end are also known as omega end. In omega-3 (*ω*-3 or n-3) and omega-6 (*ω*-6 or n-6) fatty acids, the first site of desaturation is located after the third and the sixth carbon from the omega end, respectively. Our body cannot synthesize some PUFAs, such as alpha-linolenic acid (18:3n3) and linoleic acid (18:2n6). These essential PUFAs enter our bodies only through diet. The dietary sources of n-3 PUFAs include fish oils rich in eicosapentaenoic acid (20:5n3) and docosahexaenoic acid (22:6n3), whereas the n-6 PUFA linoleic acid is mostly found in plants and vegetable oils [47].

Nowadays, there is growing evidence showing that dietary n-3 PUFAs have a variety of healthy properties such as the reduction of plasma atherogenic lipids and inflammation [16, 21, 25, 41, 47–49]. The associations between n-3 PUFAs and metabolic syndrome risk demonstrate inconsistent results [50]. Several cross-sectional and case-control studies have indicated that plasma/serum n-3 PUFAs were significantly higher in healthy subjects compared with those in patients with MetS, while some studies have suggested opposite and null associations [51]. Meanwhile, Guo et al. showed that higher circulating n-3 PUFAs were significantly associated with decreased MetS risk [52]. A study by Kim et al. demonstrated that in healthy individuals the level of long-chain n-3 PUFAs is positively correlated with insulin sensitivity [53]. A decrease in the level of PUFAs has been established in patients with T2DM and diabetic retinopathy. It was found that the development of insulin resistance is preceded by a reduction of essential n-3 PUFAs in the cell membranes [6]. There are several reasons why n-3 PUFAs are important in the pathogenesis and prevention of MetS. PUFAs perform a structural function, being important components of the cell membrane and determining its physical and chemical properties [10, 16]. The efficiency of glucose transport and expression of many receptors depend on the composition and ratio of PUFAs in the cell membrane [17]. Also, PUFAs are the precursors for inflammatory and pro-resolving lipids mediators' synthesis [29, 32]. The imbalance between the synthesis of inflammatory and pro-resolving lipids mediators determines the development of chronic inflammation in MetS. Furthermore, we will summarize the main molecular mechanisms underlying the ability of n-3 PUFAs to prevent and/or ameliorate insulin resistance and inflammation in MetS.

### 3.1. Polyunsaturated Fatty Acids and Glucose Transport

The identification of a causal relationship between the composition of fatty acids of cell membranes and MetS pathogenesis significantly contributes to an understanding of the main pathophysiological mechanisms of the disease.

Polyunsaturated fatty acids affect the fundamental properties of the cell membrane, including its fluidity, elasticity, receptor expression activity, the functionality of embedded proteins, and signal transmission through lipid rafts, which leads to changes in cell signaling and modification of gene expression [54, 55]. The length and degree of the FA chain unsaturation have a profound effect on the physical and chemical properties of cell membranes [48]. The ratio of polyunsaturated to saturated fatty acids determines the membrane flexibility, which affects the efficiency of glucose transport using insulin-independent glucose transporters (GLUTs) and insulin-dependent GLUT4 [54].

GLUT1 is a monomeric protein with 12 transmembrane helical segments [56]. One molecule GLUT1 covers an area of about 17 molecules of a phosphatidylcholine bilayer with saturated fatty acids (SFAs), which requires a high membrane flexibility for pore formation. GLUT4 is inserted into the membrane of intracellular vesicles, which demands the flexibility of the vesicular membrane. The GLUT4 containing vesicles take part in a fusion process with the cell membrane. The increased flexibility of the membrane provides a smooth bending of the cell membrane bilayer and the fused pores formation [54]. Thus, decreased membrane flexibility causes a reduction in all Class 1 glucose transporters which, in turn, reduces the glucose flux and increases the plasma glucose concentration. Therefore, high membrane flexibility is a crucial factor in glucose transport. Changes in the fatty acid composition of membranes will result in disturbance in the physicochemical properties of the bilayer, such as flexibility and fluidity. Tighter membrane packaging due to increased saturated fatty acids in it leads to a reduction in the capacity for GLUT4 glucose transport [54].

A number of other studies have also revealed that an increase in the level of saturated fatty acids in the cell membrane is associated with a growth in blood glucose level and the development of insulin resistance [13, 14]. The important role of PUFAs in maintaining glucose-insulin homeostasis is confirmed by many studies [13–15, 21, 25, 47, 49, 51, 53]. Comprehensive evidence shows that diet n-3 PUFAs can improve insulin signal transduction in adipocytes, affecting in turn the insulin-stimulated glucose uptake through the regulation of the expression or the translocation of the GLUT4 [11]. In vitro studies have found that adipocytes from n-3 PUFAs-depleted rats had lower basal and insulin-stimulated glucose incorporation, while cultured adipocytes supplemented with fish oil increased levels of GLUT4 and GLUT1 [41]. González-Périz et al. [11] reported that feeding with a marine n-3 PUFAs-enriched diet improved insulin resistance in association with an increased expression of Irs-1 and Glut4mRNA in the adipose tissue of genetically obese ob/ob mice. The above indicates the huge importance of n-3 PUFAs in the development and regulation of components in the MetS, such as insulin resistance and glucose tolerance.

### 3.2. Polyunsaturated Fatty Acids and Inflammation

Numerous studies have suggested that MetS, like its downstream sequelae of atherosclerotic cardiovascular disease and T2DM, is largely an inflammatory disease [24]. A chronic, low-grade inflammatory state caused by obesity leads to metabolic alterations responsible for multiple organ damage [57, 58]. This metabolic dysfunction could determine clinical conditions such as hypertension, hypercholesterinemia, and insulin resistance [40]. The contribution of inflammation to insulin resistance has been widely studied, and immunological changes occurring in various tissues are thought to be etiological factors affecting the development of insulin resistance [58]. A characteristic of obese people is a chronic, low-grade inflammation state promoted by the release of many inflammatory mediators by the adipose tissue and, more importantly, by infiltrating macrophages. PUFAs and their oxidized metabolites are important participants of the inflammatory processes of MetS [13, 15, 27, 28]. Understanding the molecular mechanisms of the participation of PUFAs and their metabolites in the pathogenesis of MetS will contribute to the development of new diagnostic methods and targeted therapy for this disease.

#### 3.2.1. Specialized Pro-Resolving Mediators

Inflammation is a complex, multifactorial adaptive process with different periods of development. Inflammation is a natural reaction to harmful irritants, such as bacterial infections, virus infections, and tissue damage. This is a host's defensive reaction in which immune and endothelial cells and proinflammatory mediators are attracted to eliminate inflammatory agents, clear damaged cells and tissues, and initiate tissue repair. This response, when properly functioning, is self-limiting and leads to the cessation of the inflammatory response and a return to homeostasis, a process called the resolution of inflammation [16, 48]. Resolution of inflammation is now known to be an active process involving the activation of negative feedback mechanisms, such as anti-inflammatory cytokine secretion, reduction in receptor expression, activation of regulatory cells, and production of pro-resolving lipid mediators [57]. However, when acute inflammation is intense or prolonged, the resolution process is not successful, which leads to excessive tissue damage and ultimately resulting in chronic inflammation [16]. Many studies have confirmed that unresolved inflammation is the main mechanism for the pathogenesis of MetS [15, 16, 24, 48, 57, 58].

**Figure 1:**
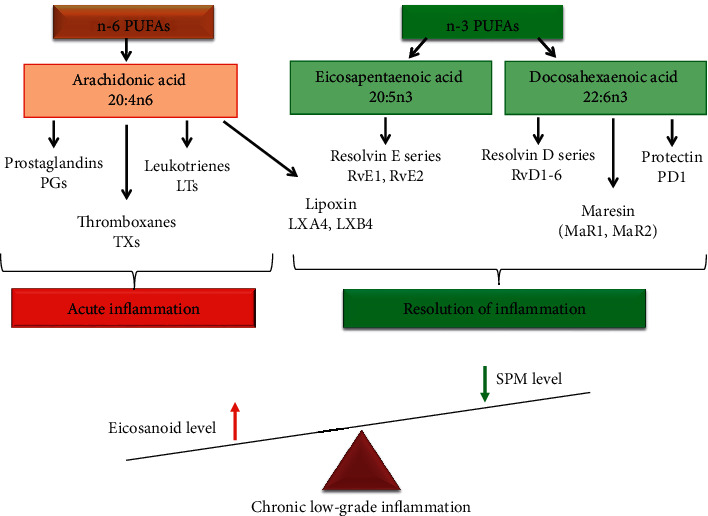
PUFA pathway and role of lipid mediators in the development and resolution of inflammation.

PUFAs are a source of synthesis of inflammatory and pro-resolving lipid mediators. The major substrate for the synthesis of inflammatory lipid mediators is arachidonic acid (20:4n6) (see Figure 1). The high content of 20:4n6 provides a direct link with inflammation since 20:4n6 released from cell membrane phospholipids acts as a substrate for cyclooxygenase (COX), lipoxygenase (LOX), and cytochrome P450 enzymes [29]. Eicosanoids are important regulators and mediators of acute inflammatory processes and include prostaglandins (PGs), thromboxanes (TBs), and leukotrienes (LTs). Many anti-inflammatory therapies, such as nonsteroidal anti-inflammatory drugs and COX inhibitors, target arachidonic acid metabolism [29, 35, 59, 60].

Eicosapentaenoic acid (20:5n3) and docosahexaenoic acid (22:6n3) from the n-3 PUFAs family are a source of synthesizing specialized pro-resolving mediators (SPMs): maresins, resolvins, and protectins (see Figure 1) [32–35]. SPMs are a class of cell compounds generated at a later stage of the inflammation and initiate the resolution of the inflammatory process [33, 34, 61]. SPMs actively facilitate the resolution stage of acute inflammation unlike eicosanoids, which mainly act during the first stage of inflammation. A balanced n-6 : n-3 PUFAs ratio (where 1 : 1 to 2 : 1 is optimal) is important for homeostasis and normal development throughout the lifespan. High n-6 PUFA intake in the Western diet increases the n-6 : n-3 ratio to a range from 10 : 1 to 20 : 1 and may play a role in the pathogenesis of MetS and related diseases [36]. The balance between n-3 PUFAs and n-6 PUFAs determines the path of inflammatory response. The prevalence of n-6 PUFAs and the shortage of n-3 PUFAs may contribute to impaired inflammation resolution [31].

Docosahexaenoic acid- and eicosapentaenoic acid-derived SPMs are identified in the adipose tissue. At the same time, the levels of certain SPMs are markedly reduced with obesity, suggesting adipose SPM deficiency, potentially resulting in unresolved inflammation [36].

Resolvins are synthesized spontaneously from eicosapentaenoic and docosahexaenoic acids during inflammation and thus are designated as E‐series (RvE) and D‐series (RvD), respectively [32]. The anti-inflammatory effect of RvE1 is due to interaction with peroxisome proliferator-activated receptors (PPARs), which are classified as nuclear transcription factors with anti-inflammatory activity. Leukotriene B4 receptor 1 (BLT1) and G protein-coupled receptor, Chemerin Receptor 23 (ChemR23), are receptors for RvE1. RvE2 has a similar biologic effect; it regulates neutrophil chemotaxis and activates phagocytosis and proinflammatory cytokines synthesis [31, 62].

Protectins (PD) are another class of pro‐resolving molecules produced from 22:6n3 during the resolution of inflammation. Protectins are synthesized by a number of cells including brain cells, monocytes, and CD4+ lymphocytes [33]. PD1, the key representative of the protectin family, demonstrates a strong anti-inflammatory and neuroprotective effect. This mediator functioning is based on PPARs interacting and NF-*κ*B blocking [62].

An alternative process for docosahexaenoic acid oxygenation is found in human macrophages and platelets, leading to the synthesis of maresin 1 (MaR1). In addition, 13S, 14S‐epoxy‐maresin, which has important biological activities of its own, is the precursor for maresin 2 (MaR2) [36].

Lipoxins (LXs) are powerful anti-inflammatory bioregulators suppressing inflammation and activating resolution and recovery processes, in particular in MetS [34]. The substrate for LXs synthesis is arachidonic acids. Two members of the LXs family, LXA4 and LXB4, have been well studied [31]. In general, LXs are a branch of the leukotriene family. For example, their production by platelets is catalyzed by 12-LOX through converting LTA4 [32]. Unlike proinflammatory LTs, LXs act as powerful anti-inflammatory bioregulators, suppressing the inflammation and activating the processes of resolution and recovery. The result of their action is the inhibition of chemotaxis and migration of macrophages and neutrophils to the inflammatory focus, blocking of the lipid peroxidation, the activation of NF-*κ*B, and the suppression of the synthesis of proinflammatory cytokines. In addition, LXs are actively involved in functioning of macrophages that are associated with homeostasis restoration processes [32].

There is a considerable amount of evidence regarding the contribution of n‐3 PUFAs to diseases with inflammatory conditions, such as metabolic syndrome [25, 34–36, 47, 49, 51, 53]. It was reported that SPM levels reduced in metabolic syndrome as well as sensitivity to SPM of the adipose tissue [36]. Obesity reduces the levels of PD1, intermediates in the synthesis of D-series resolvins and protectins (17-HDHA), and intermediates in the maresin biosynthesis (14-HDHA) for the adipose tissues from diet- and genetically-induced obese mice [36]. One of the mechanisms resulting in a decrease in the SPM level in obesity is a change in the enzyme activities involved in biosynthesis or conversion of certain SPMs. N‐3 PUFAs supplementation increased the level of SPM in the blood of individuals with obesity and MetS. The effects of n-3 PUFAs are mediated by their ability to interfere with arachidonic acid metabolism and promote the synthesis of SPMs. The supply of n‐3 PUFAs increases the levels of resolvins, enhances resolution, and improves insulin sensitivity in an experiment with fat‐1 mice. In addition, n‐3 PUFAs prevent macrophage increase, adipokine secretion, and insulin resistance induced by a high‐fat diet. Synthetic pro-resolving lipid mediators (17-hydroxy-DHA) or n-3 PUFAs added to the treatment contributed to higher levels of pro-resolving lipid mediators in the adipose tissue, reduced inflammation, and increased insulin sensitivity [31]. N-3 PUFAs increased RvЕ-series levels in patients with MetS but did not affect RvD-series, which requires further studies into the mechanism of n-3 PUFAs influence in MetS. For instance, intraperitoneal administration of 17‐HDHA or RvD1 significantly reduced adipose inflammation and improved the glucose tolerance in diet‐induced obese mice and in db/db mice [63]. Treatment with either RvD1 or RvD2 also reduced the secretion of proinflammatory cytokines including TNF‐*α*, IL‐1*β*, and IL‐12 in the adipose tissue [64]. The MaR1 treatment improved insulin sensitivity, determined with an insulin tolerance test. MaR1 also increased adiponectin gene expression and Akt phosphorylation in the adipose tissues and attenuated adipose tissue inflammation in both ob/ob and diet‐induced obese mice [36]. PD1 treatment acutely increased the adiponectin transcripts in adipose tissue explants isolated from ob/ob mice. A potent ability to induce adiponectin expression/secretion has been demonstrated with synthetical RvD1, RvD2, and PD1 and their biosynthetic intermediate, 17‐HDHA [63].

Therefore, one of the pathogenetic mechanisms of the development of MetS is a reduction of the processes of resolving inflammation and the development of chronic, low-grade inflammatory. A decrease in the synthesis of specialized pro-resolving lipid mediators is the basis of the above disorders [65]. Thus, the anti-inflammatory effect of n-3 PUFAs in MetS can be mediated through the regulation of the SPM synthesis.

#### 3.2.2. Toll-Like Receptor 4

The inflammatory process observed in individuals with metabolic syndrome differs from the classical inflammatory response and this type of inflammation characterized by a chronic, low-intensity reaction [58]. The toll-like receptor 4 (TLR4) signaling pathway is acknowledged as one of the main triggers of the obesity-induced inflammatory response [57]. TLR4 plays a significant role in the pathogenesis of inflammation mediated by insulin resistance in MetS [57]. Toll-like receptors, including TLR4, are type 1 transmembrane proteins with three domains: (1) extracellular domain with leucine-rich repeats (LRRs) responsible for ligand recognition; (2) transmembrane domain; and (3) intracellular toll/interleukin-1 receptor (TIR) domain. These provide signal transmission from the cell surface to adapter proteins. TLR4 was the first TLR reported in humans; it is expressed in innate immune cells, including monocytes, macrophages, and dendritic cells, as well as in other cell types, such as adipocytes, enterocytes, and muscle cells. TLR4 is a membrane-associated receptor involved in lipid recognition [66]. TLRs are activated both by the influence of endogenous ligands and by the participation of lipids—cholesterol, SFAs, and oxidized forms of phospholipids [67].

Humans with type I diabetes exhibit greater expression of TLR4 in the cellular membrane in monocytes. Individuals with T2DM show increased cellular membrane levels of TLR4 in blood monocytes, as well as a higher concentration of IL-1, IL-6, IL-8, and TNF in serum. Similarly, TLR4 is more highly expressed in blood mononuclear cells and in the abdominal subcutaneous white adipose tissue of obese and diabetic individuals [57, 68].

**Figure 2:**
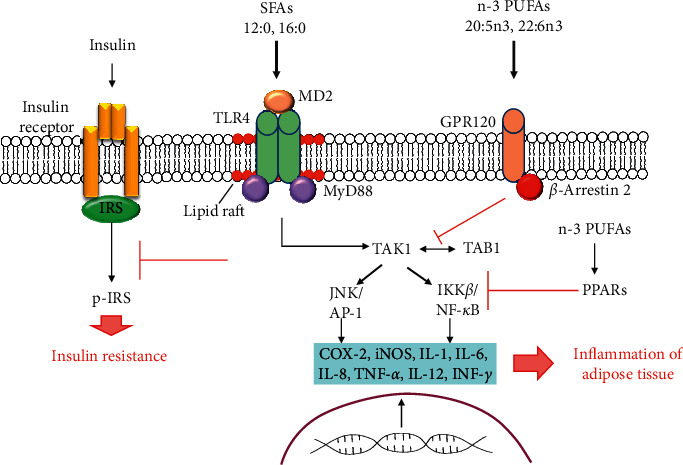
The role of TLRs and FAs in the signaling mechanisms of inflammation in the adipose tissue and insulin resistance. The saturated fatty acids (SFAs) act as nonmicrobial TLR4 agonists or indirectly promote the TLR4 activation, triggering its inflammatory response and inflammation of the adipose tissue. Inflammatory signaling caused by saturated fatty acids via TLR4/MD-2 inhibits the phosphorylation of the insulin receptor, leading to the development of insulin resistance. GPR120 activation induced by n-3 PUFA leads to a decrease in the activity of IKK-*β*/NF-*κ*B and JNK/AP-1 signaling pathways, which reduces the expression of proinflammatory genes. The anti-inflammatory properties of PPARs are achieved by inhibiting nuclear factor-kappa B (NF-*κ*B). N-3 PUFAs directly interact with PPARs and modulate the expression of proinflammatory genes.

Lipids from foods change the expression of TLRs by cells [69]. On the one hand, SFAs activate the TLR4 signaling pathway (see Figure 2). Among the SFAs, lauric acid (12 : 0) and palmitic acid (16 : 0) had the strongest activation capacity through TLR4 [69]. On the other hand, TLRs can be inhibited by PUFAs [70]. Consumption of n-3 PUFAs, particularly 22:6n3, is associated with anti-inflammatory and cardioprotective effects. It is believed that the use of n-3 PUFAs is associated with anti-inflammatory activity due to inhibition of arachidonic acid metabolism [71]. The molecular effect of n-3 PUFAs, especially 20:5n3 and 22:6n3, on inflammatory-response modulation are based on the ability of these PUFAs to inhibit the expression of inflammatory genes, such as COX-2, iNOS, and IL-1 in macrophages [72]. PUFAs of the n-3 family reduce the activation of the NF-*κ*B transcription factor pathway that is induced by various agonists [70].

Other mechanisms modulate the inflammatory response by fatty acids based on binding G protein-coupled receptor 120 (GPR120) [66]. GPR120 is a free fatty acid 4 receptor (FFAR4), and GPR120 activation induced by n-3 PUFA leads to *β*-arrestin 2 recruitment to the plasma membrane where this protein binds to GPR120 (see Figure 2) The GPR120/*β*-arrestin 2 complex is internalized into the cytoplasmic compartment where this complex binds to the TAK1-binding protein (TAB1). This process impairs the association between TAB1 and the kinase activated by the growth factor beta (TAK1) and, consequently, results in reduced TAK1 activation and decreases the activity of the IKK-*β*/NF-*κ*B and JNK/AP-1 signaling pathways. The mitigation of TAK-1 activation by n-3 PUFAs leads to the reduced expression of TNF-*α* and IL-6 genes with proinflammatory actions [17, 57].

One more important molecular mechanism that is associated with the n-3 PUFA effects concerns their capacities to bind to PPARs [62]. Three isoforms of PPARs are known: PPAR*α* (NR1C1), PPAR*β*/*δ* (NR1C2), and PPAR*γ* (NR1C3). PPARs are involved in the regulation of inflammatory reactions and lipid metabolism. The anti-inflammatory properties of PPARs are mainly achieved by inhibiting nuclear factor-kappa B (NF-*κ*B) which, in turn, is the proinflammatory nuclear transcription factor [73]. The interactions between PPARs, NF-*κ*B, and toll-like receptors (TLRs) are of great interest. Along with the anti-inflammatory mechanism of action of PPARs, the proinflammatory activity of some isoforms of PPARs is also being studied. For example, PPAR*γ* is considered a mediator of interactions between dendritic and T cells in the development of type 2 (or T2) inflammation [73]. N-3 PUFAs directly interact with PPARs and, therefore, modulate the expression of genes that are involved in lipid metabolism and the inflammatory response [57]. Anti-inflammatory effects of 20:5n3 and 22:6n3 on this signaling pathway can occur due to diminished nicotinamide adenine dinucleotide phosphate (NADPH) oxidase activity, which leads to lower TLR4 recruitment for lipid rafts and TLR4 dimerization [16]. Also, another possible mechanism of action of the n-3 PUFA concerns the capacity of incorporating 22:6n3 into the plasma membrane, which can lead to reduced TLR4 translocation for lipid rafts formation [74, 75]. The variety of molecular mechanisms in lipids and TLR4 signaling pathway interaction indicates the complexity of the pathogenesis of MetS and associated diseases.

### 3.3. Polyunsaturated Fatty Acids and Plasmalogens

Permanent exogenous use of PUFA is a necessary condition for maintaining immune-metabolic homeostasis. The profile of fatty acids that are present in the Western diet consists of a high level of saturated fatty acids and trans fatty acids. While the total consumption of marine and plant n-3 polyunsaturated fatty acids in contemporary society is significantly reduced [7, 76].

Another reason for PUFAs reduction is deterioration in the plasmalogen synthesis [77]. Plasmalogens are a subclass of phospholipids characterized by having a vinyl ether bond linking the fatty aldehyde to the glycerol molecule in the 1-position and a fatty acyl bond in the 2-position. The sn-1 position consists of palmitic acid (16 : 0), stearic acid (18 : 0), or oleic acid (18 : 1) carbon chains, and the head group is usually either ethanolamine or choline. Thus, there are two main types of plasmalogens: ethanolamine plasmalogens and choline plasmalogens. The sn-2 position is generally occupied by PUFAs, specifically arachidonic acid or docosahexaenoic acid [78, 79].

The highest concentrations of plasmalogens are found in the brain, red blood cells, skeletal muscle, and spermatozoa and can represent as much as 18–20% of the total phospholipids in cell membranes [78, 79]. Plasmalogens are either derived from dietary sources and/or are synthesized mainly in the liver and gastrointestinal epithelium. Plasmalogens are not only important structural phospholipids in the cell membranes but they are also reservoirs of secondary messages and mediators of membrane dynamics and involved in membrane fusion, ion transport, cholesterol efflux, membrane-bound enzyme activity, and diffusion of signal-transduction molecules [80].

Secondary deficiency of plasmalogens triggered by their synthesis reduction or their degradation growth is associated with metabolic and inflammatory disorders such as cardiac diseases and diabetes mellitus [77]. The specificity of choline plasmalogens as a sensitive biomarker of an atherogenic state was confirmed. On the one hand, positive correlations of the choline plasmalogen content with serum adiponectin concentration and high-density lipoproteins (HDL), and on the other hand, inverse relationships with waist circumference, including triacylglycerides and low-density lipoproteins (LDL) content, have been identified. Reduced levels of ethanolamine plasmalogens in plasma have been shown to be also closely associated with cardiovascular, metabolic, and cancer diseases [81]. The content of plasmalogens is relatively stable in all lipoprotein fractions. However, the correlation between the levels of choline plasmalogens and HDL is stronger than that between the levels of ethanolamine plasmalogens and HDL. In the study by Pietiläinen et al., a decrease in the level of plasmalogens in adipocyte membranes in obese twins was established compared with metabolically healthy twins. Conversely, plasmalogen levels increase in trained people and dietary patients [82]. At the same time, it was found that the level of plasmalogens increases in the liver of rats receiving a high-fat diet [83].

The adaptation of the phospholipid composition of cells to exogenous lipid changes has been verified [83]. The compensatory response to a decrease in the plasmalogen level is the regulation of the level of phosphatidylethanolamine [84]. However, with plasmalogen deficiency, the total amount of PUFAs in phosphatidylethanolamine remains constant in human fibroblasts and in the brains of mice. Plasmalogens have been noted to play an important role as neuroprotectors and modulators of the signaling mechanisms of cell membranes [85]. Plasmalogens also act as endogenous antioxidants, protecting lipids and lipoproteins from oxidative stress [86]. This can be attributed to the fact that the hydrogen atoms adjacent to the vinyl ether bond are more susceptible to oxidation, protecting PUFAs from it that are found in the sn-2 position of the glycerol residue. Plasmalogen oxidation products are not capable of further initiation of lipid peroxidation processes. Another important function of plasmalogens is their participation in cell metabolism and transmembrane transport of FAs. The presence of PUFAs in the side chains of plasmalogens preconfigures their significant depositing function [77]. Cholesterol esterification depends on the level of plasmalogens. So, for example, the cells characterized by plasmalogen deficiency demonstrated a lower level of esterified cholesterol and a higher level of free and total cholesterol [84].

Therefore, the important role of plasmalogens as modulators of signaling mechanisms in protecting cells from lipid peroxidation and participation in PUFA metabolism has been made clear. However, the exact biological functions of plasmalogens and the underlying molecular mechanisms still remain to be discovered [52, 55].

## 4. Free Fatty Acids and Metabolic Syndrome Risk

Free fatty acids (FFAs), or nonesterified fatty acids (NEFAs), in circulating plasma are derived from the ingestion of dietary fat or from the triglycerides stored in adipose tissue that are distributed to cells to serve as fuel for muscle contraction and systemic metabolism [87]. As FAs are insoluble in water, they are transported by binding to plasma albumin. FFAs can be taken up from circulating plasma by all mitochondria-containing cells, and they are metabolized by *β*-oxidation [17]. FFAs carry out many important biological functions in the body, and they are a source of energy, signal molecules, and structural components of cell membranes [17]. FFAs are involved in the pathogenesis of insulin resistance and subsequent development of metabolic syndrome [12, 17, 87]. Chronic energy imbalance can trigger adipocyte hypertrophy, endoplasmic reticulum stress, and mitochondrial dysfunction, which lead to the systemic release of FFAs [17, 88–90]. When plasma FFA levels rise, as occurs in obesity, a lipotoxicity state is induced, which induce activation of different cell responses: oxidative stress, apoptosis, and inflammation [17]. Consequently, FFAs play a highly important role in the association between obesity and insulin resistance.

### 4.1. FFAs and Mitochondria

There is an interesting hypothesis that IR is associated with the development of mitochondrial dysfunction [87, 90]. Lipid degradation occurs in mitochondria. On top of that, the normal functioning of mitochondria provides glucose-stimulated insulin secretion from *β*-cells of the pancreas. Initially, the theories have suggested that impaired mitochondrial function leads to impaired *β*-oxidation of lipids, which is accompanied by the accumulation of FFAs in peripheral tissues (lipotoxicity theory) [91]. The accumulation of lipid metabolites brings about the activation of kinases involved in the disruption of insulin signaling at the level of insulin receptor substrate 1 (IRS-1). In skeletal muscles, insulin signaling pathway disorder is accompanied by a decrease in the production of GLUT4 and glucose uptake by cells. In this case, an improvement in insulin sensitivity can be achieved by enhancing the *β*-oxidation of lipids. This theory was supported by studies that proved an increase in the rate of *β*-oxidation of lipids to be followed by protection against the development of IR [17].

Nevertheless, the early stages of obesity and IR development are characterized by an increase in *β*-oxidation of lipids. Besides, an impairment of fat oxidation results in higher insulin production. Therefore, mitochondrial dysfunction in skeletal muscles cannot be the only reason for the development of IR [92].

An alternative explanation of the relationship between mitochondria and insulin resistance is focused on the production of a reactive oxygen species (ROS) by mitochondria as a result of excess accumulation of FFAs in them [92]. Oxidative stress is known to be a pathogenetic component of chronic inflammation development and IR [88]. An oxidized redox environment can induce insulin resistance by directly affecting the protein involved in glucose uptake [89].

On the other hand, changes in redox cell homeostasis have been argued to step up the activity of the serine-/threonine-sensitive stress kinases that inhibit the transmission of insulin signals, inducing the development of IR [93, 94].

**Figure 3:**
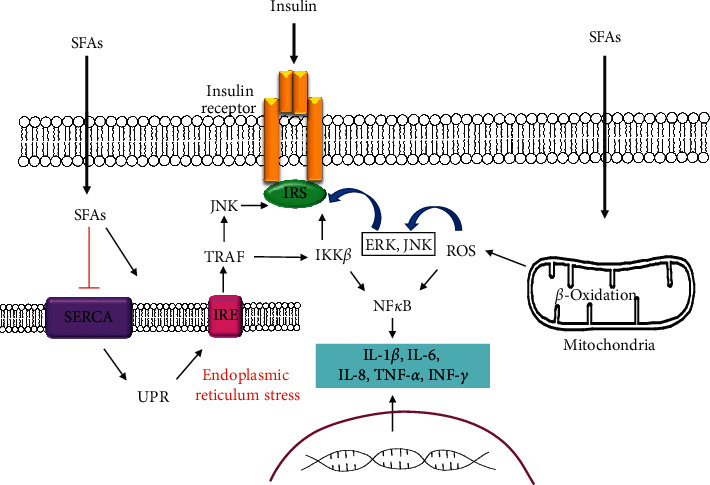
FFA-induced insulin resistance through endoplasmic reticulum stress and oxidative stress. A high level of FFA induces an increase in the production of ROS by mitochondria and the formation of oxidative stress. ROS stimulates NF-*κ*B, which promotes the synthesis of L-1*β*, IL-6, and TNF-*α*. These inflammatory cytokines contribute to obesity-associated local inflammation and directly induce insulin resistance. In response to the enhanced level of FFAs and other nutrients in fats, adipose cells can develop signs of ER stress. A decrease in SERCA expression promotes the development of ER stress. UPR triggers the activation of IRE. Activation of IRE induces interaction with TRAF protein, which stimulations activation of IKK*β* and JNK kinases. Its reaction can phosphorylate the IRS, thus blocking insulin signaling. JNK and IKK*β* also lead to NF-*κ*B activation and the development of inflammation.

Oxidative stress also can stimulate the activation of transcriptional factors, such as nuclear factor-kappa B (NF-*κ*B), activator protein 1 (AP-1), and hypoxia-inducible factor 1 (HIF-1), which promote the synthesis of inflammatory cytokines (IL-1*β*, IL-6, and TNF-*α*) (see Figure 3). These inflammatory cytokines contribute to obesity-associated local inflammation and directly induce insulin resistance. Also, chronic prolonged FFAs excess is the cause of pancreatic *β*-cells dysfunction. In addition, FFAs inhibit insulin gene expression and induce apoptosis in these cells [17].

Although discussing the role of mitochondrial skeletal muscle dysfunction in the pathogenesis of IR and type 2 diabetes is still underway [93], it is generally accepted that a mitochondrial defect does occur in these diseases. The connection between IR and mitochondrial dysfunction of liver cells, visceral, and subcutaneous adipose tissue has been proved [95]. Moreover, in the mitochondria of individuals suffering from obesity and type 2 diabetes, ATP synthesis is reduced, which correlates with the accumulation of FFAs and inhibition of insulin-stimulated glucose utilization.

### 4.2. FFAs and Endoplasmic Reticulum Stress

Results of numerous studies establish that dysregulation of the endoplasmic reticulum (ER) function contributes to the development of MetS [96, 97]. Mitochondria are known to be both functionally and structurally associated with ER [97]. Obviously, the changes of the structure and function of these organelles can serve as a trigger for the development of metabolic homeostasis disorders [96]. ER is involved in maintaining Ca2+ homeostasis and participates in maturation and expression of membrane and secretion proteins. Cell stress conditions that increase ER demand and entail an overload of its functional capacity cause a series of alterations known as “endoplasmic reticulum stress.” Under these conditions, the ER activates a compensatory mechanism called the “unfolding protein response” (UPR), which attempts to restore the homeostasis of ER functions. With the stressful effects lasting for a long time, ER stress results in cell death (apoptosis) [17, 98].

UPR triggers activation of inositol-requiring endoribonuclease enzyme (IRE) (see Figure 3). The activation of IRE induces interaction with TRAF protein, which stimulations activation of IKK*β* and JNK kinases. Its reaction can phosphorylate IRS, thus blocking insulin signaling. In response to the enhanced level of FFAs and other nutrients in fats, adipose cells can develop signs of ER stress [17]. ER stress produces insulin resistance mainly through JNK activation. JNK activity has been detected to be elevated in animal models of obesity, and JNK isoforms 1 and 2 deletion protects mice from insulin resistance induced by a fat-rich diet. Experimental evidence indicates that, on the one hand, JNK phosphorylates serine IRS-1, and on the other, it phosphorylates IKK*β*, which leads to NF-*κ*B activation and to inflammation development [96]. Remarkably, that change in expression of sarco/endoplasmic reticulum Ca2+ ATPase (SERCA), which has calcium elimination from the cytosol and returns it to the ER as their function, is associated with ER stress and subsequently with insulin resistance. The treatment of people with diabetes mellitus by rosiglitazone, an antidiabetic drug, increased SERCA expression, thus restoring the pump expression reduction observed in diabetic patients with altered hyperglycemia [17]. This way, the decrease in SERCA expression promotes the development of ER stress, with JNK ensuing activation, which desensitizes the insulin signal, thus generating a state of insulin resistance and contributing to chronic metabolic deterioration.

### 4.3. FFAs as Ligands for FFAR

FFAs serve not only as energy sources but also as natural ligands for a group of orphan G protein-coupled receptors (GPCRs) termed free fatty acid receptors (FFARs) [99]. The GPCR superfamily is the largest one in the human genome and encompasses some subfamilies (Gq, Gi, Gs, and G12/13) [100]. These receptors respond to various ligands and, therefore, are involved in the pathogenesis of many diseases, e.g., MetS, and are the target for more than half of pharmaceutical products [101–106]. There are four main members of FFAR family: FFAR1 (GPR40), FFAR2 (GPR43), FFAR3 (GPR41), and FFAR4 (GPR120 and GPR84) (see Table 1) [75].

FFAR1 expression was revealed in neurons and in pancreas *β*-cells [99]. FFAR2 and FFAR3 are common in leukocytes and adipose tissues. Besides, FFAR3 is also expressed by pancreas cells, in the sympathetic nervous system and vessel plain muscles [100]. FFAR4 is expressed in adipocytes, the intestinal tract, macrophages, and in the central nervous system [105]. There are other specific receptors for FFA: GPR119 and GRP84. GPR119 is expressed in intestinal endocrine cells and pancreatic *β*-cells and activates the synthesis of GLP-1 and insulin. GPR84 is expressed in the spleen, thymus, leukocytes, and macrophages [99]. Long- and medium-chain length fatty acids are endogenous ligands for FFAR1, FFAR4, and GPR84. FFAR2 and FFAR3 are activated by short-chain FAs. FFAR2 is capable of binding with Gq and Gi proteins, whereas FFAR3 binds only with Gi. FFAR4 is activated by n-3 or n-6 PUFAs [99]. Thus, each FFAR can act as an FFA sensor with selectivity for a particular FFA carbon chain length derived from food or food-derived metabolites. FFARs have been reported to have physiological functions such as facilitation of insulin and incretin hormone secretion, adipocyte differentiation, anti-inflammatory effects, neuronal responses, and taste preferences [106]. Dysfunction of FFARs underlies the pathogenesis of many metabolic diseases, such as MetS and diabetes mellitus.

It has been found that FFAR4 acts as an anti-inflammatory receptor in proinflammatory macrophages and mature adipocytes. Signaling of FFAR4 activated by n-3 PUFAs inhibits TLR signaling and TNF-*α*-induced inflammatory responses. FFAR4 dysfunction leads to obesity and glucose intolerance in humans and mice [107]. Many results strongly support that FFAR4-mediated anti-inflammatory effects reduce the infiltration of proinflammatory macrophages into the adipose tissue and improve insulin sensitivity [102].

The activation of FFAR1 signaling enhances glucose-stimulated insulin secretion (GSIS) directly via stimulation of insulin secretion from pancreatic *β* cells and indirectly via the production of incretin hormones. Also, the activation of FFAR1 signaling reduces the expression of inflammatory cytokines such as TNF-*α* and IL-8. It has been shown that *α*-linolenic (18:3n3) and oleic (18:1n9) acids improve insulin resistance in obesity and type 2 diabetes [108].

There is some scientific evidence that short-chain fatty acids (SCFAs) are a substantial modulator of MetS inflammation [109]. SCFAs are the end products of metabolic fermentation of dietary fibers by gut microbiota. FFAR2 is a receptor for SCFAs and is expressed in enteroendocrine cells, adipose tissues, and pancreatic *β*-cells [99]. Dietary fiber intake reduces the risk of obesity, diabetes, inflammatory bowel disease, colon cancer, and cardiovascular disease. SCFAs supplementation with a high-fat diet improved insulin sensitivity and increased energy expenditure in a mouse model of diet-induced obesity [110, 111]. SCFAs are involved in intestinal immune homeostasis due to their role in regulating T cell polarization and differentiation. In human monocytes, SCFAs decrease the production of TNF-*α* and increase the production of PGE2 [109]. Activation of FFAR2 by SCFAs regulates metabolic disorders, increases energy expenditure, and preferentially enables fat consumption by inhibition of insulin signaling in adipose tissues. The expression of FFAR2 in neutrophils and mononuclear cells regulates intestinal homeostasis and inflammation. In light of this evidence, regulation FFAR2 expression and/or high fiber consumption may be a potential target for therapeutic intervention of MetS.

FFAR3 is also a receptor for SCFAs. FFAR3 is widely expressed in enteroendocrine cells, adipose tissues, the peripheral nervous system, peripheral blood mononuclear cells, monocytes, and macrophages [99]. FFAR3 expression in intestinal epithelial cells enhances the synthesis of proinflammatory mediators through extracellular signal-regulated kinase 1/2 and p38 MAPK [100]. Since these pathways help to protect against bacterial infection, FFAR3 can stimulate acute inflammatory reactions in the intestine that have beneficial effects on host homeostasis [112]. Thus, FFAR 3 can exhibit proinflammatory properties.

## 5. Conclusion

The wide phenotypic heterogeneity of MetS and its complex pathogenesis make it difficult to identify a therapeutic target. This syndrome is considered as a cluster of pathogenetically related conditions caused by metabolic disorders and the development of chronic, low-grade inflammation. In this review, we examined the molecular mechanisms of the development of MetS driven by impaired lipid metabolism. PUFAs and FFAs have been shown to play an important role in both the pathogenesis and treatment of MetS. Fatty acids perform structural, energy, signaling, and immunoregulatory functions. These FAs properties underlie the pathogenetic mechanisms of glucose transport disturbance, the development of IR and chronic inflammation, the formation of oxidative stress, and mitochondrial dysfunction in MetS. Correction in lifestyle and nutrition is considered as the main way to minimize complications caused by an imbalance in the body between saturated and polyunsaturated fatty acids. At the same time, there are controversial data about the therapeutic efficacy of dietary n-3 PUFAs in MetS [50]. SPMs have shown potent pro-resolving actions in different disease models, including MetS [61]. SPM-based therapeutics could be one of the most optimistic treatments for MetS. Further studies are needed to detail the mechanisms of FA participation and their oxidized metabolites in the development of inflammation and pathogenesis of MetS.

## Figures and Tables

**Table 1:** Family of FFARs and their ligands.

Free fatty acid receptors	Expression	Ligand
FFAR1 (GPR40)	Nerve cells, immune cells, adipocytes, pancreatic *β*-cells, smooth muscle cells of respiratory tract	Medium-chain fatty acids and long-chain fatty acids
FFAR2 (GPR43)	Nerve cells, immune cells, adipocytes, pancreatic *β*-cells, intestinal epithelial cells, airway epithelial cells	Short-chain fatty acids
FFAR3 (GPR41)	Nerve cells, immune cells, adipocytes, pancreatic *β*-cells, intestinal epithelial cells, airway epithelial cells	Short-chain fatty acids
FFAR4 (GPR120)	Nerve cells, adipocytes, immune cells, pancreatic *β*-cells, cells of the intestine	Medium-chain fatty acids and long-chain fatty acids

## References

[1] Nilsson P. M., Tuomilehto J., Rydén L. (2019). The metabolic syndrome—what is it and how should it be managed?,. *European Journal of Preventive Cardiology*.

[2] Wang H. H., Lee D. K., Liu M., Portincasa P., Wang D. Q.-H. (2020). Novel insights into the pathogenesis and management of the metabolic syndrome. *Pediatric Gastroenterology, Hepatology & Nutrition*.

[3] Ranasinghe P., Mathangasinghe Y., Jayawardena R., Hills A. P., Misra A. (2017). Prevalence and trends of metabolic syndrome among adults in the asia-pacific region: a systematic review. *BMC Public Health*.

[4] Sigit F. S., Tahapary D. L., Trompet S. (2020). The prevalence of metabolic syndrome and its association with body fat distribution in middle-aged individuals from Indonesia and the Netherlands: a cross-sectional analysis of two population-based studies. *Diabetology & Metabolic Syndrome*.

[5] Lee M.-K., Han K., Kim M. K. (2020). Changes in metabolic syndrome and its components and the risk of type 2 diabetes: a nationwide cohort study. *Scientific Reports*.

[6] Denisenko Y., Novgorodtseva T., Zhukova N. (2015). Metabolic syndrome: modification of the fatty acid composition and glucose-insulin homeostasis. *British Journal of Medicine and Medical Research*.

[7] Silva Figueiredo P., Carla Inada A., Marcelino G. (2017). Fatty acids consumption: the role metabolic aspects involved in obesity and its associated disorders. *Nutrients*.

[8] Grgurevic I., PodrugStéphanie Monnerie K., Comte B. (2020). Metabolomic and lipidomic signatures of metabolic syndrome and its physiological components in adults: a systematic review. *Scientific Reports*.

[9] Surowiec I., Noordam R., Bennett K. (2019). Metabolomic and lipidomic assessment of the metabolic syndrome in Dutch middle-aged individuals reveals novel biological signatures separating health and disease. *Metabolomics*.

[10] Novgorodtseva T. P., Karaman Y. K., Zhukova N. V., Lobanova E. G., Antonyuk M. V., Kantur T. A. (2011). Composition of fatty acids in plasma and erythrocytes and eicosanoids level in patients with metabolic syndrome. *Lipids in Health and Disease*.

[11] González-Périz A., Horrillo R., Ferré N. (2009). Obesity-induced insulin resistance and hepatic steatosis are alleviated by *ω*3 fatty acids: a role for resolvins and protectins. *The FASEB Journal*.

[12] Weijers R. N. (2012). Lipid composition of cell membranes and its relevance in type 2 diabetes mellitus. *Current Diabetes Reviews*.

[13] Martínez-Fernández L., Laiglesia L. M., Huerta A. E., Martínez J. A., Moreno-Aliaga M. J. (2015). Omega-3 fatty acids and adipose tissue function in obesity and metabolic syndrome. *Prostaglandins & Other Lipid Mediators*.

[14] Tremblay A. J., Després J.-P., Piché M.-È. (2004). Associations between the fatty acid content of triglyceride, visceral adipose tissue accumulation, and components of the insulin resistance syndrome. *Metabolism*.

[15] Phinney S. D. (2005). Fatty acids, inflammation, and the metabolic syndrome. *The American Journal of Clinical Nutrition*.

[16] Calder P. C. (2017). Omega-3 fatty acids and inflammatory processes: from molecules to man. *Biochemical Society Transactions*.

[17] Vázquez-Jiménez J. G., Roura-Guiberna A., Jiménez-Mena L. R., Olivares-Reyes J. A. (2017). Role of free fatty acids on insulin resistance. *Gaceta Médica de México*.

[18] Lark D. S., Fisher-Wellman K. H., Neufer P. D. (2012). High-fat load: mechanism(s) of insulin resistance in skeletal muscle. *International Journal of Obesity Supplements*.

[19] Cena H., Calder P. C. (2020). Defining a healthy diet: evidence for the role of contemporary dietary patterns in health and disease. *Nutrients*.

[20] Glass C. K., Olefsky J. M. (2012). Inflammation and lipid signaling in the etiology of insulin resistance. *Cell Metabolism*.

[21] Lepretti M., Martucciello S., Burgos Aceves M., Putti R., Lionetti L. (2018). Omega-3 fatty acids and insulin resistance: focus on the regulation of mitochondria and endoplasmic reticulum stress. *Nutrients*.

[22] Koehrer P., Acar N., Saab S. (2013). Polyunsaturated fatty acids and plasmalogens in diabetics. *Investigative Ophthalmology & Visual Science*.

[23] Kytikova O. Y., Novgorodtseva T. P., Antonyuk M. V., Gvozdenko T. A. (2019). Plasmalogenes in the pathophysiology and therapy of age-specific diseases. *Advances in Gerontology*.

[24] Reddy P., Lent-Schochet D., Ramakrishnan N., Mclaughlin M., Jialal I. (2019). Metabolic syndrome is an inflammatory disorder: a conspiracy between adipose tissue and phagocytes. *Clinica Chimica Acta*.

[25] Lambert C., Cubedo J., Padró T. (2017). Phytosterols and omega 3 supplementation exert novel regulatory effects on metabolic and inflammatory pathways: a proteomic study. *Nutrients*.

[26] Kim O.-K., Jun W., Lee J. (2015). Mechanism of ER stress and inflammation for hepatic insulin resistance in obesity. *Annals of Nutrition and Metabolism*.

[27] Grandl G., Wolfrum C. (2018). Hemostasis, endothelial stress, inflammation, and the metabolic syndrome. *Seminars in Immunopathology*.

[28] Han M. S., White A., Perry R. J. (2020). Regulation of adipose tissue inflammation by interleukin 6. *Proceedings of the National Academy of Sciences*.

[29] Gilroy D. W., Bishop-Bailey D. (2019). Lipid mediators in immune regulation and resolution. *British Journal of Pharmacology*.

[30] Headland S. E., Norling L. V. (2015). The resolution of inflammation: principles and challenges. *Seminars in Immunology*.

[31] Kytikova O., Novgorodtseva T., Denisenko Y., Antonyuk M., Gvozdenko T. (2019). Pro-resolving lipid mediators in the pathophysiology of asthma. *Medicina*.

[32] Serhan C. N., Chiang N., Dalli J. (2018). New pro-resolving n-3 mediators bridge resolution of infectious inflammation to tissue regeneration. *Molecular Aspects of Medicine*.

[33] Aursnes M., Tungen J. E., Vik A. (2014). Total synthesis of the lipid mediator PD1n-3 DPA: configurational assignments and anti-inflammatory and pro-resolving actions. *Journal of Natural Products*.

[34] Doğan E. S. K., Doğan B., Fentoğlu Ö, Kırzıoğlu F. Y. (2019). The role of serum lipoxin A4 levels in the association between periodontal disease and kwonlic syndrome. *Journal of Periodontal & Implant Science*.

[35] Barden A. E., Mas E., Croft K. D., Phillips M., Mori T. A. (2015). Specialized proresolving lipid mediators in humans with the metabolic syndrome after n-3 fatty acids and aspirin. *The American Journal of Clinical Nutrition*.

[36] Kwon Y. (2020). Immuno‐resolving ability of resolvins, protectins, and maresins derived from omega‐3 fatty acids in metabolic syndrome. *Molecular Nutrition & Food Research*.

[37] Xiang Y., Zhou W., Duan X. (2019). Metabolic syndrome, and particularly the hypertriglyceridemic-waist phenotype, increases breast cancer risk, and adiponectin is a potential mechanism: a case-control study in Chinese women. *Front Endocrinol (Lausanne)*.

[38] Lee J. H., Lee K. S., Kim H. (2020). The relationship between metabolic syndrome and the incidence of colorectal cancer. *Environmental Health and Preventive Medicine*.

[39] Wang Y., Tu R., Yuan H. (2020). Associations of unhealthy lifestyles with metabolic syndrome in Chinese rural aged females. *Scientific Reports*.

[40] Ju K. O., Antonjuk M. V., Gvozdenko T. A., Novgorodceva T. P. (2018). Metabolic aspects of the relationship of obesity and bronchial asthma. *Obesity and Metabolism*.

[41] Amatruda M., Ippolito G., Vizzuso S., Vizzari G., Banderali G., Verduci E. (2019). Epigenetic effects of n-3 LCPUFAs: a role in pediatric metabolic syndrome. *International Journal of Molecular Sciences*.

[42] Halter J. B., Musi N., McFarland Horne F. (2014). Diabetes and cardiovascular disease in older adults: current status and future directions. *Diabetes*.

[43] Yach D., Stuckler D., Brownell K. D. (2006). Epidemiologic and economic consequences of the global epidemics of obesity and diabetes. *Nature Medicine*.

[44] WHO—World Health Organization (2020). World Health Organization obesity and overweight fact sheet. https://www.who.int/en/news-room/fact-sheets/detail/obesity-and-overweight.

[45] Jiang J., Cai X., Pan Y. (2020). Relationship of obesity to adipose tissue insulin resistance. *BMJ Open Diabetes Research & Care*.

[46] Harayama T., Riezman H. (2018). Understanding the diversity of membrane lipid composition. *Nature Reviews Molecular Cell Biology*.

[47] Tortosa-Caparrós E., Navas-Carrillo D., Marín F., Orenes-Piñero E. (2017). Anti-inflammatory effects of omega 3 and omega 6 polyunsaturated fatty acids in cardiovascular disease and metabolic syndrome. *Critical Reviews in Food Science and Nutrition*.

[48] Calder P. C. (2020). n‐3 PUFA and inflammation: from membrane to nucleus and from bench to bedside. *Proceedings of the Nutrition Society*.

[49] Iwase Y., Kamei N., Takeda-Morishita M. (2015). Antidiabetic effects of omega-3 polyunsaturated fatty acids: from mechanism to therapeutic possibilities. *Pharmacology & Pharmacy*.

[50] Radcliffe J. E., Thomas J., Bramley A. L. (2016). Controversies in omega-3 efficacy and novel concepts for application. *Journal of Nutrition & Intermediary Metabolism*.

[51] Albracht-Schulte K., Kalupahana N. S., Ramalingam L. (2018). Omega-3 fatty acids in obesity and metabolic syndrome: a mechanistic update. *The Journal of Nutritional Biochemistry*.

[52] Guo X.-f., Li X., Shi M., Li D. (2017). n-3 polyunsaturated fatty acids and metabolic syndrome risk: a meta-analysis. *Nutrients*.

[53] Kim Y.-S., Xun P., He K. (2015). Fish consumption, long-chain omega-3 polyunsaturated fatty acid intake and risk of metabolic syndrome: a meta-analysis. *Nutrients*.

[54] Weijers R. N. M. (2016). Membrane flexibility, free fatty acids, and the onset of vascular and neurological lesions in type 2 diabetes. *Journal of Diabetes & Metabolic Disorders*.

[55] Wong S. W., Kwon M.-J., Choi A. M. K., Kim H.-P., Nakahira K., Hwang D. H. (2009). Fatty acids modulate toll-like receptor 4 activation through regulation of receptor dimerization and recruitment into lipid rafts in a reactive oxygen species-dependent manner. *Journal of Biological Chemistry*.

[56] Salas-Burgos A., Iserovich P., Zuniga F., Vera J. C., Fischbarg J. (2004). Predicting the three-dimensional structure of the human facilitative glucose transporter Glut1 by a novel evolutionary homology strategy: insights on the molecular mechanism of substrate migration, and binding sites for glucose and inhibitory molecules. *Biophysical Journal*.

[57] Rogero M., Calder P. C. (2018). Obesity, inflammation, toll-like receptor 4 and fatty acids. *Nutrients*.

[58] Minihane A. M., Vinoy S., Russell W. R. (2015). Low-grade inflammation, diet composition and health: current research evidence and its translation. *British Journal Of Nutrition*.

[59] Peebles R. S. (2019). Prostaglandins in asthma and allergic diseases. *Pharmacology & Therapeutics*.

[60] Marcone S., Evans P., Fitzgerald D. J. (2016). 15-deoxy-Δ12,14-prostaglandin J2 modifies components of the proteasome and inhibits inflammatory responses in human endothelial cells. *Frontiers in Immunology*.

[61] Serhan C. N., Levy B. D. (2018). Resolvins in inflammation: emergence of the pro-resolving superfamily of mediators. *Journal of Clinical Investigation*.

[62] Kytikova O. Y., Perelman J. M., Novgorodtseva T. P. (2020). Peroxisome proliferator-activated receptors as a therapeutic target in asthma. *PPAR Research*.

[63] Neuhofer A., Zeyda M., Mascher D. (2013). Impaired local production of proresolving lipid mediators in obesity and 17-HDHA as a potential treatment for obesity-associated inflammation. *Diabetes*.

[64] Clària J., Dalli J., Yacoubian S., Gao F., Serhan C. N. (2012). Resolvin D1 and resolvin D2 govern local inflammatory tone in obese fat. *The Journal of Immunology*.

[65] Morshedzadeh N., Saedisomeolia A., Djalali M., Eshraghian M. R., Hantoushzadeh S., Mahmoudi M. (2019). Resolvin D1 impacts on insulin resistance in women with polycystic ovary syndrome and healthy women. *Diabetes & Metabolic Syndrome: Clinical Research & Reviews*.

[66] Sidletskaya K., Vitkina T., Denisenko Y. (2020). The role of toll-like receptors 2 and 4 in the pathogenesis of chronic obstructive pulmonary disease. *International Journal of Chronic Obstructive Pulmonary Disease*.

[67] Ruysschaert J. M., Lonez C. (2015). Role of lipid microdomains in TLR-mediated signalling. *Biochimica et Biophysica Acta*.

[68] Prajapati B., Jena P. K., Rajput P. (2014). Understanding and modulating the toll like receptors (TLRs) and NOD like receptors (NLRs) cross talk in type 2 diabetes. *Current Diabetes Reviews*.

[69] Huang S., Rutkowsky J. M., Snodgrass R. G. (2012). Saturated fatty acids activate TLR-mediated proinflammatory signaling pathways. *Journal of Lipid Research*.

[70] Hwang D. H., Kim J. A., Lee J. Y. (2016). Mechanisms for the activation of Toll-like receptor 2/4 by saturated fatty acids and inhibition by docosahexaenoic acid. *European Journal of Pharmacology*.

[71] Lalia A. Z., Lanza I. R. (2016). Insulin-sensitizing effects of omega-3 fatty acids: lost in translation?,. *Nutrients*.

[72] Li Y., Deng S. L., Lian Z. X. (2019). Roles of toll-like receptors in nitroxidative stress in mammals. *Cells*.

[73] Huang D., Zhao Q., Liu H., Guo Y., Xu H. (2016). PPAR-*α* agonist WY-14643 inhibits LPS-induced inflammation in synovial fibroblasts via NF-kB pathway. *Journal of Molecular Neuroscience*.

[74] Shaikh S. R., Kinnun J. J., Wassall S. R. (2015). How polyunsaturated fatty acids modify molecular organization in membranes: insight from NMR studies of model systems. *Biochimica et Biophysica Acta*.

[75] Wassall S. R., Leng X., Canner S. W., Pennington E. R., Kinnun J. J., Cavazos A. T. (2018). Docosahexaenoic acid regulates the formation of lipid rafts: a unified view from experiment and simulation. *Biochimica et Biophysica Acta (BBA)—Biomembranes*.

[76] Saklayen M. G. (2018). The global epidemic of the metabolic syndrome. *Current Hypertension Reports*.

[77] Kytikova O. Y., Novgorodtseva T. P., Antonyuk M. V. (2019). Molecular targets of fatty acid ethanolamides in asthma. *Medicina*.

[78] Honsho M., Abe Y., Fujiki Y. (2017). Plasmalogen biosynthesis is spatiotemporally regulated by sensing plasmalogens in the inner leaflet of plasma membranes. *Scientific Reports*.

[79] Messias M. S. F., Mecatti G. C., Priolli D. G., Carvalho O. (2018). Plasmalogen lipids: functional mechanism and their involvement in gastrointestinal cancer. *Lipids in Health and Disease*.

[80] Su X., Wang J., Sinclair A. J. (2019). Plasmalogens and Alzheimer’s disease: a review. *Lipids in Health and Disease*.

[81] Barchuk M., Dutour A., Ancel P. (2020). Untargeted lipidomics reveals a specific enrichment in plasmalogens in epicardial adipose tissue and a specific signature in coronary artery disease. *Arteriosclerosis, Thrombosis, and Vascular Biology*.

[82] Pietiläinen K. H., Sysi-Aho M., Rissanen A. (2007). Acquired obesity is associated with changes in the serum lipidomic profile independent of genetic effects—a monozygotic twin study. *PLoS One*.

[83] Dean J. M., Lodhi I. J. (2018). Structural and functional roles of ether lipids. *Protein & Cell*.

[84] Wallner S., Orsó E., Grandl M., Konovalova T., Liebisch G., Schmitz G. (2018). Phosphatidylcholine and phosphatidylethanolamine plasmalogens in lipid loaded human macrophages. *PLoS One*.

[85] West A., Zoni V., Teague W. E. (2020). How do ethanolamine plasmalogens contribute to order and structure of neurological membranes?,. *The Journal of Physical Chemistry B*.

[86] Broniec A., Żądło A., Pawlak A. (2017). Interaction of plasmenylcholine with free radicals in selected model systems. *Free Radical Biology and Medicine*.

[87] Suiter C., Singha S. K., Khalili R., Shariat-Madar Z. (2018). Free fatty acids: circulating contributors of metabolic syndrome. *Cardiovascular & Hematological Agents in Medicinal Chemistry*.

[88] Ghosh A., Gao L., Thakur A., Siu P. M., Lai C. W. K. (2017). Role of free fatty acids in endothelial dysfunction. *Journal of Biomedical Science*.

[89] Alicka M., Marycz K. (2018). The effect of chronic inflammation and oxidative and endoplasmic reticulum stress in the course of metabolic syndrome and its therapy. *Stem Cells International*.

[90] Montgomery M. K., Turner N. (2015). Mitochondrial dysfunction and insulin resistance: an update. *Endocrine Connections*.

[91] Ježek P., Jabůrek M., Holendová B., Plecitá-Hlavatá L. (2018). Fatty acid-stimulated insulin secretion vs. lipotoxicity. *Molecules*.

[92] Holloszy J. O. (2013). “Deficiency” of mitochondria in muscle does not cause insulin resistance. *Diabetes*.

[93] Martin S. D., McGee S. L. (2014). The role of mitochondria in the aetiology of insulin resistance and type 2 diabetes. *Biochimica et Biophysica Acta*.

[94] Zhenyukh O., González-Amor M., Rodrigues-Diez R. R. (2018). Branched-chain amino acids promote endothelial dysfunction through increased reactive oxygen species generation and inflammation. *Journal of Cellular and Molecular Medicine*.

[95] Bouderba S., Sanz M. N., Sánchez-Martín C. (2012). Hepatic mitochondrial alterations and increased oxidative stress in nutritional diabetes-prone Psammomys obesus model. *Experimental Diabetes Research*.

[96] Rieusset J. (2017). Role of endoplasmic reticulum-mitochondria communication in type 2 diabetes. *Advances in Experimental Medicine and Biology*.

[97] Maiers J. L., Malhi H. (2019). Endoplasmic reticulum stress in metabolic liver diseases and hepatic fibrosis. *Seminars in Liver Disease*.

[98] Ly L. D., Xu S., Choi S.-K. (2017). Oxidative stress and calcium dysregulation by palmitate in type 2 diabetes. *Experimental & Molecular Medicine*.

[99] Kimura I., Ichimura A., Ohue-Kitano R., Igarashi M. (2020). Free fatty acid receptors in health and disease. *Physiological Reviews*.

[100] Miyamoto J., Hasegawa S., Kasubuchi M., Ichimura A., Nakajima A., Kimura I. (2016). Nutritional signaling via free fatty acid receptors. *International Journal of Molecular Sciences*.

[101] Yonezawa T., Kurata R., Yoshida K., Murayama M. A., Cui X., Hasegawa A. (2013). Free fatty acids-sensing G protein-coupled receptors in drug targeting and therapeutics. *Current Medicinal Chemistry*.

[102] Congreve M., de Graaf C., Swain N. A., Tate C. G. (2020). Impact of GPCR structures on drug discovery. *Cell*.

[103] Pujol J. B., Christinat N., Ratinaud Y., Savoia C., Mitchell S. E., Dioum E. H. M. (2018). Coordination of GPR40 and ketogenesis signaling by medium chain fatty acids regulates beta cell function. *Nutrients*.

[104] Zhang Q., Yang H., Li J., Xie X. (2016). Discovery and characterization of a novel small-molecule agonist for medium-chain free fatty acid receptor g protein-coupled receptor 84. *Journal of Pharmacology and Experimental Therapeutics*.

[105] Milligan G., Shimpukade B., Ulven T., Hudson B. D. (2017). Complex pharmacology of free fatty acid receptors. *Chemical Reviews*.

[106] Bartoszek A., Moo E. V., Binienda A. (2020). Free fatty acid receptors as new potential therapeutic target in inflammatory bowel diseases. *Pharmacological Research*.

[107] Miyamoto J., Kasubuchi M., Nakajima A., Kimura I., Milligan G., Kimura I. (2016). Anti-inflammatory and insulin-sensitizing effects of free fatty acid receptors. *Free Fatty Acid Receptors. Handbook of Experimental Pharmacology*.

[108] Oliveira V. (2015). Diets containing *α*-linolenic (*ω*3) or oleic (*ω*9) fatty acids rescues obese mice from insulin resistance. *Endocrinology*.

[109] Hu J., Lin S., Zheng B., Cheung P. C. K. (2018). Short-chain fatty acids in control of energy metabolism. *Critical Reviews in Food Science and Nutrition*.

[110] Sun M., Wu W., Liu Z., Cong Y. (2017). Microbiota metabolite short chain fatty acids, GPCR, and inflammatory bowel diseases. *Journal of Gastroenterology*.

[111] Sivaprakasam S., Prasad P. D., Singh N. (2016). Benefits of short-chain fatty acids and their receptors in inflammation and carcinogenesis. *Pharmacology & Therapeutics*.

[112] Kim M. H. (2013). Short-chain fatty acids activate GPR41 and GPR43 on intestinal epithelial cells to promote inflammatory responses in mice. *Gastroenterology*.

